# Mr. Rolfe, on Yeast in Typhus

**Published:** 1801-06

**Authors:** W. D. Rolfe

**Affiliations:** Dispensary House, North Street


### *To the Editors of the Medical and Phyſical Journal.*

Gentlemen,

THROUGH the medium of your intereſting Journal, the uſe of yeaſt in typhus and putrid fevers has ſeveral times been ſpoken of; could my practice add a mite to the ſtock of information, the duty I owe to my profeſſion would induce me to exert every ability in my power. If the following caſes are worthy of your inſertion, they are much at your ſervice.

I was requeſted to viſit Margaret Jackſon, aged 42, who had been ill for ſome time with putrid fever; ſhe was then delirious, her tongue, teeth, and lips were covered with a black fur, and ſhe had alſo a violent diarrhœa. I thought this a favourable opportunity for trying the yeaſt: I told the daughter, the time for medicine to be of ſervice was gone by, but if ſhe would attend punctually to my directions, I could lay down a plan that might poſſibly relieve her mother. The girl, anxious for her mother's recovery, (having left her place of

ſervice

ſervice to nurſe her) promiſed ſhe would be punctual. Being a truly diſtreſſed family, I gave them money to purchaſe yeaſt, which I continued to do through their illneſs, as their ſituation would otherwiſe exclude them from getting a ſufficient quantity, and the caſe might be imperfect for the want of it. The woman continued taking it, diluted with water, until ſhe was perfectly recovered, without any other medical aſſiſtance, and there alſo ſeemed leſs proſtration of ſtrength when the fever was over, than I ever found in a patient before, conſidering the ſeverity of the diſeaſe and the melancholy circumſtances of the patient. A few days afterwards the huſband came home ill, and in twenty-four hours he was delirious; the ſymptoms nearly the ſame as the wife, excepting the huſband being very coſtive, which I, however, totally diſregarded, as I did the wife's diarrhœa, leaving Nature to take her own courſe. The huſband being fully convinced of the effect of the yeaſt on his wife, took it immediately, and continued it through the whole courſe of his delirium, rejecting every thing elſe until he was perfectly recovered. At the ſame time one of the daughters was taken ill, and by a deal of exertion, got down a conſiderable quantity, by which ſhe got quite well. Before this daughter got well the eldeſt was taken ill, and purſued the ſame plan, by which means ſhe alſo was perfectly recovered.

I am, &c.

*Diſpenſary Houſe, North Street,*
*May* 8, 1801.

W. D. ROLFE.

---

*State of the Patients in the Briſtol Diſpenſary, from 1ſt of April to the 1ſt of May,* 1801.

